# A comparative study on biopharmaceutical function of curcumin and miR-34a by multistimuli-responsive nanoniosome carrier: *In-vitro* and *in-vivo*

**DOI:** 10.3389/fmolb.2022.1043277

**Published:** 2022-10-17

**Authors:** Najmeh Alsadat Abtahi, Seyed Morteza Naghib, Fateme Haghiralsadat, Mohammadmahdi Akbari Edgahi, Esfandyar Askari

**Affiliations:** ^1^ Nanotechnology Department, School of Advanced Technologies, Iran University of Science and Technology, Tehran, Iran; ^2^ Medical Nanotechnology and Tissue Engineering Research Center, Yazd Reproductive Sciences Institute, Shahid Sadoughi University of Medical Sciences, Yazd, Iran; ^3^ Biomaterials and Tissue Engineering Research Group, Department of Interdisciplinary Technologies, Breast Cancer Research Center, Motamed Cancer Institute, ACECR, Tehran, Iran

**Keywords:** curcumin, gene expression, coadminstration, nanopartcicles, chemotherapy, niosome

## Abstract

This research conducted a comparative study on nanoscaled niosomal structures consisting of Tween-80, Tween-60, cholesterol, and dioleoyl-3-trimethylammonium propane (DOTAP). Thin-film hydration technique was used for the preparation and entrapment of curcumin and miRNA in niosomal formulations for enhancing the stability and delivery rate of the agents. Herein, the influence of Tween-80, Tween-60, cholesterol, and DOTAP on the entrapment efficiency (EE%) of curcumin and the physicochemical properties of the carrier are fully discussed. The optimum engineered formulation resulted in a positive charge of +11.23 mV, high EE (100%), smooth surface, spherical shape, small diameter (90 nm), and good stability in physiological buffers. Also, an accelerated cellular uptake, as well as drug release in PBS (pH 7.4, 37°C) after 72 h, were observed. The cytotoxic activity of curcumin (Cur)/miR-34a-loaded nanoparticles was determined by the MTT assay*.* The results displayed an improved cytotoxic activity of Cur-niosome towards cancer cells compared to free-dispersed Cur. The uptake of Cur-loaded niosome by A280s and A280cp-1 cancer cell lines faced 2.5 folds drop in the concentration compared to its free form. Generally, Cur-niosome exhibits a significant accumulation of superior anti-cancer properties. Likewise, the cytotoxicity of miR-34a-niosome against tumor cells was higher in comparison with its free form. The anti-cancer effects of the gene/drug delivery were investigated in the 4T1 xenografted Balb/C mouse tumor model. According to the *in vitro* and *in vivo* results, gene delivery from the modified niosome nanoparticles was distinctly greater than Cur delivery. Therefore, it was concluded that encapsulation of genes in the nano-niosomal delivery system is a promising procedure for the treatment of cancer cells.

## 1 Introduction

While an alternation occurs in biological systems, there is a tendency for cells to lose their normal function and begin to do abnormal activities. Herein, p53 is a tumor suppressor protein that is highly critical in cell response to stresses such as DNA injury and oncogenes activation. Many tumor malignancies arise when their functionality is faced with problems (Boutelle and Attardi, 2021). P53 can modify specific gene expression captured *via* apoptosis, improve DNA repair, and prevent angiogenesis (Merlin et al., 2021).

As stated by many studies, the most frequent miRNA which is induced *via* this tumor suppressor protein is the miR-34 group (Sargolzaei et al., 2020). The anti-cancer mechanism of this category of miRNAs is based on the suppression of tumors which approves their potential for anti-tumoral purposes (Najminejad et al., 2019). Generally, microRNAs or miRNAs are small-sized RNAs with non-coding ability that strongly influence tumorigenesis, apoptosis, cell proliferation, and differentiation. They can alter gene expression in the post-transcriptional stage (Tricoli and Jacobson, 2007). Several pieces of research displayed a significant reduction in the level of specific miRNAs which operate as tumor suppressors within cancerous areas. From experimental findings, miRNAs with such properties are categorized in 15a, 16-1, 143, let-7, 145, and 34 (Pidíkova et al., 2020). The miR-34 family involves important mediators with tumor-suppressing ability. Various documents indicate that through ectopic expression of miR-34s, many factors, including proliferation, invasion, metastasis, and epithelial-to-mesenchymal transition, are eliminated. Besides, an initial viral vector in miR-34 delivery is critical (Flood et al., 2019). Two commonly used materials are niosomes and liposomes to deliver miRNA to the cancer site. In recent years, the use of these vesicular carriers in systemic delivery applications has received a lot of interest because of their acceptable entrapment efficacy, reduced side effects, increased drug solubility, long-term blood circulation, and capability to target a specific spot (Abtahi et al., 2022).

Curcumin (Cur) has also attracted scientific research in terms of delivery applications. Cur is a yellowish natural diphenolic compound derived from the rhizome of Curcuma Longa, turmeric, which has been widely used as a spice or as a medicine in Asian countries (India, China, and Indonesia) for the treatment of a variety of illnesses, including stomach disorders, skin diseases, bile formation problems, anorexia, rhinitis, sinusitis, cough, diabetic lesions, liver function problems, and rheumatism.

Polyphenol was revealed to have numerous pharmacological effects in 1970, including antibacterial, anti-inflammatory, antioxidant, and anti-tumor capabilities (Chauhan et al., 2019; He et al., 2021; Hassan et al., 2022; Moghadam et al., 2022). Cur’s anti-cancer mechanism on malignant cells is based on complicated molecular signaling pathways, including estrogen receptor, proliferative pathways, and human epidermal growth factor 2 receptor, and eventually, induces apoptosis, according to recent reviews in this field (Wang Y et al., 2016; Kunnumakkara et al., 2017; Talib et al., 2018; Farhadihosseinabadi et al., 2019; Song et al., 2019). Moreover, Cur is a regulator of the p53 protein in breast cancer (Talib et al., 2018). Cur has been shown to regulate several cell signaling pathways, including cell survival (c-IAP1, cFLIP, Bcl-xL, Bcl-2, XIAP), cell multiplication (cyclin D1, c-myc), caspase stimulation (caspase-3, 8, 9), protein kinase (JNK, Akt, and AMPK), mitochondrial, death receptor (DR4, DR5), and tumor inhibitor (Yen et al., 2019).

Despite its positive characteristics, Cur free use in clinical cancer therapy is limited because of its low water solubility, poor oral absorption, and rapid metabolism. As a result, scientists have been looking for novel ways to address the aforementioned shortcomings (Chendil et al., 2004; Mozafari et al., 2015; Wong et al., 2019). Nanotechnology helps to solve these issues by establishing cancer diagnostics and treatment (Meng et al., 2020; Khan et al., 2021; Yi et al., 2021). The creation of drug delivery systems (DDSs) aids in the distribution of low-affinity medications to tissues and cells, improving treatment effectiveness and reducing systemic adverse effects at the target location (Meng et al., 2020; Eftekhari et al., 2021). The excellent efficacy of encapsulating, managing drug release, improving drug solvability, conveying hydrophilic and hydrophobic pharmaceuticals, and longer blood circulation given by these carriers have made vesicular medication administration popular in recent decades (Wu et al., 2021; Almansob et al., 2022). Due to its ability to capture both hydrophobic and hydrophilic medicines within its bilayer *via* non-polar and core cavity regions, niosomes have attracted increasing scientific interest as valuable DDSs in recent years. Niosome vesicles are non-ionic multilamellar or unilamellar surfactants ranging from 10 to 1,000 nm and are non-immunogenic, biodegradable, and biocompatible. Since the non-ionic surfactants used for the preparation of niosome are cheaper than phospholipids, they also have taken a larger part in nanotechnology. Niosomes are being studied for the delivery of drugs like Roxithromycin, Bovine Serum Albumin, and Doxorubicin, as well as siRNA/miRNA delivery. As a result, various nanocarrier formulations for Cur have been introduced, including liposomes and niosomes. Similarly, manifold documents on the nano-potential carriers for transporting other therapeutic medicines, such as miRNA, have been published.

Curcumin and miR-34a were therefore incorporated into novel niosomal nanoformulations in the current work to boost cancer cell targeting while lowering harmful effects on healthy cells. To do this, we created and improved multiple nano-drug formulations, described them chemically and physically, and then tested their biological effects on normal and cancer cell lines. Co-delivery refers to the use of curcumin and miR-34a in the same carrier for medication delivery. Curcumin and miR-34a packaged in niosomes were delivered together, and the impact was studied *in vitro* and *in vivo* (Nikniaz et al., 2021).

## 2 Materials and methods

### 2.1 Materials

Herein, Tween-80 (T-80) and Tween-60 (T-60) (DaeJung Chemicals & Metals, Seoul, South Korea), DMSO (3-[4,5-dimethylthiazol-2-yl]-2), curcumin, cholesterol, and MTT (3-[4,5-dimethylthiazol-2-yl]-2,5 diphenyl tetrazolium bromide) (Sigma-Aldrich, Missouri, United States), and DOTAP (1,2-dioleoyl-3-trimethylammonium-propane), were acquired for this study. Human ovary cancer cells (Pasteur Institute, Tehran, Iran), FBS, antibiotics, PBS, cell culture medium (RPMI 1640), trypsin, and sodium pyruvate were also provided for the experiment (Gibco, New York, United States). All the chemicals were of analytical grade and no extra purification process was applied for salts, chemicals, and solvents, except in particular cases. The order synthesis of miR-34a primer was forward, CTT​GAA​CTC​CTG​GGG​CCT​GAA​G; reverse, GCC​AAA​GAA​ACA​CTC​ACA​GCT, and Eurofins Genomics Ebersberg were utilized for this reason.

### 2.2 Preparation of niosomal delivery system

In the preparation of niosomes, thin-film hydration technique was employed. Cholesterol and T-80 were first computed precisely and then dissolved in 100 ml of chloroform. Then, a rotating flash evaporator (Ultrasonics GmbH, Heidolph, Germany) was conducted to form a thin lipid at low pressures. Afterward, the obtained film underwent 3 ml of PBS for the hydration process at pH 7.4 and 60°C. To decrease the mean size of vesicles, a microtip probe sonicator was utilized to operate the sonication process of hydrated lipids for 30 min. Then, the niosome formula was monitored to detect the physical properties. In the next step, for achieving enhanced stability of niosome formula cationic lipid, DOTAP, was added. Besides, forming a thin layer on a rotating evaporator resulted in eliminating organic solvent at 45°C. For obtaining niosome suspension, the hydration of the thin layers was achieved by adding PBS at pH 7.4 and 60°C for 45 min. Finally, the niosomal solution was sonicated using (Ultrasonics GmbH, Hielscher, Germany) for 15 min to reduce the average particle size.

### 2.3 Characterization of nanoparticles

To assess the overall characteristics of niosomes, including particle size, zeta potential, and poly-dispersity index (PDI), dynamic light scattering (DLS) analysis was conducted by Brookhaven Corp Instrument (Brookhaven Instruments; Holtsville; NY: United States). Likewise, Atomic force microscopy (AFM), Scanning electron microscopy (SEM), and Transmission electron microscopy TEM (AFM5100N, HITACHI) were conducted to assess the morphology of the gene and drug-loaded niosome. The resulting data and mean values were used for triplicate measurement.

### 2.4 Encapsulation efficiency of curcumin

To measure the encapsulation efficiency, first, the unencapsulated drugs were subtracted from the loaded niosomes by immersing them into the dialysis cellulose membrane tubing (12 kDa MWCO) against 4°C PBS solution. Afterward, the niosomes were lysed using isopropanol and a UV-Vis spectrophotometer, (T80+, PG Instruments, United Kingdom) 429 nm wavelength, was utilized to determine the encapsulation efficiency according to the following equation:

$$Encapsulation\;Efficiency\;\left(\%\right)=\frac{The\;quantity\;of\;Cur\;capsulized\;inside\;niosomes}{The\;total\;quantity\;of\;Cur\;added\;}\times 100$$

### 2.5 Drug release

To assess the released quantity of Cur from niosomes, a 12 kDa MWCO dialysis tube was employed (Shaker et al., 2015). For this purpose, 10 mM PBS was used as the buffer solution, and samples were held at 25, 37, and 42°C for 72 h, at pH values of 5.5, 6.5, and 7.4. Afterward, all samples were individually suspended into the dialysis bag and stirred constantly. At each interval, aliquots of PBS were taken and the same volume of PBS was inserted. Ultimately, UV-Vis spectrophotometry was employed to determine the release rate (Shaker et al., 2015).

### 2.6 Leakage stability

To assess the effect of long-lasting storage on the leakage behavior of miRNA from nanocomplexes, samples were held at 4°C for 2 months. Afterward, nanometer the stability of all samples was recorded using spectrophotometric at 260 nm.

### 2.7 Physical stability

The physical characteristic of Cur-loaded niosome, parameters such as the size of particles, PDI, residual quantity, and zeta potential were obtained using DLS analysis through Zeta PALS zeta potential and particle size analyzer (Br particle size distribution, Brookhaven Instruments, New York, United States) at intervals of 15, 30, and 60 days.

### 2.8 Particle morphology

The morphology of nanoparticles was examined by subjecting a drop of the nanoparticle solution to negative staining with 1% (w/v) phosphor tungstic sodium solution and AFM, SEM, and TEM were performed.

### 2.9 Cell culture

To culture cells, first, human ovarian cancer A2780s and A2780cp-1 cells (Iranian Biological Resource Center, Tehran, Iran) were preserved as monolayer cultures in a mixture of Ham and RPMI-1640 medium (Ino Clon, Tehran, Iran) augmented with 2 mM of GlutaMAX™-I (100X), 10% FBS, and 1 mg/ml penicillin/streptomycin (Gibco, Massachusetts, United States). The MCF-10A cell line (Iranian Biological Resource Center, Tehran, Iran), a non-tumorigenic human breast epithelial cell line, was cultured in DMEM/F12 Ham’s mixture enriched with 1 mg/ml of penicillin/streptomycin, 5% horse serum, 2 mM of Gluta MAX™-I (Gibco, Massachusetts, US), 100 ng/ml cholera toxin, 10 μg/ml insulin, EGF 20 ng/ml, and hydrocortisone 0.5 μg/ml (Sigma-Aldrich, Missouri, United States). The MCF-10A cells were utilized as the control group in all tests.

### 2.10 Cytotoxicity and cell growth

The cytotoxic activity of Cur-loaded nanoparticles was clarified by MTT assay (Sigma-Aldrich, Missouri, US) (Wang J et al., 2016; Xu et al., 2016). Briefly, cells were seeded in 96-well plates at 10,000 cells per well. After a day, 200 μL of a new media containing sequential dilutions of several formulations, including niosomal Cur, free Cur solution, and free niosome, was inserted into the cells. Then, all of the prepared samples were incubated for 1, 2, and 3 days (s). Next, in each 96-well plate, 20 ml of MTT (5 mg/ml in PBS) was added and followed by incubation at 37°C for 3 h for further verification. Next, the medium was carefully separated, instead, to suspend the produced formazan crystals, each well received 180 ml of dimethyl sulfoxide. Records of optical density in each well were conducted using EPOCH Microplate Spectrophotometer (synergy HTX, BioTek, Vermont, US) at the wavelengths of 570 and 630 nm.

The cytotoxic activity of samples was shown as the value of the Inhibitory Concentration (IC_50_), which represents the least concentration of drug to inhibit 50% of cell growth compared to the control. The Cur IC_50_ levels as single drugs were estimated by Graph Pad Prism 6. The test was performed in triplicate.

### 2.11 Assessment of cellular uptake

A2780s and A2780cp-1 cells were seeded in 6-well plates at a concentration of 50,000 cells/well and kept in an incubator for 1 day to enable cell attachment. Next, different concentrations of Cur were added to wells. Then, cells were incubated for another 3 h followed by washing thrice with cold PBS and fixed with a mixed solution of methanol and citric acid (3:1) (Sigma-Aldrich, Missouri, United States). After staining with 4′,6-diamidino-2-phenylindole (DAPI, 0.125 μg/ml, Thermo Fisher Scientific, Massachusetts, US), cell images were obtained using fluorescent microscopy (BX61, Olympus, Japan) (Balakrishnan et al., 2009). The test was done only once.

### 2.12 Apoptosis assay

In apoptosis of cells, they were subjected to different samples as free Cur, free miRNA, niosomal Cur, and niosomal miRNA at IC_50_ concentration for all of them, and annexin V-FITC/PI double staining (Sigma-Aldrich, Missouri, United States) was performed to determine the dead cells. First, MCF-7 cells were seeded into 6-well plates at a concentration of 100,000 cells/well and incubated for 24 h. Afterward, for detaching the cells, 0.25% trypsin/EDTA was added to the wells and then they were centrifuged at 1,500 rpm for 3 min. Next, the pellet was dissolved in the ice-cold PBS at pH 7.4 and then 3 μl of Annexin V-FITC was inserted into the suspension. Ultimately, 3 μl of propidium iodide (PI) stock solution was inserted into the cells, followed by incubating for 30 min on ice. BD FACSCalibur instrument was used to analyze the flow cytometry and detect the stained cells.

### 2.13 *In vivo* experiments

To perform *in-vivo* examination according to NIH and IACUC guidelines, 35 six- to 8-week female BALB/c mice (Pasteur Institute, Tehran, Iran) with 20–25 g bodyweight were purchased and kept in a sterilized condition. First, each mouse was treated with 5 × 10^6^ 4T1 cells subcutaneously into the right flank. Next, they were randomly divided into 4 groups of 5 mice when the tumor reaches 100 mm^3^. Free Cur and miRNA with 2.5 mg/kg and niosomal Cur and miRNA with 10 μg per mouse were injected within the tail vein of each group of mice. For the control group, normal saline was injected. The injections were repeated every 3 days up until 12 days and at each interval, the tumor size and bodyweight of each mouse were measured. All mice were sacrificed on the 21st day. The tumor size was measured according to the equation (Abtahi et al., 2022):

$$V\;\left({mm}^{3}\right)=\frac{{LW}^{2}}{2}$$

Where V represents the volume of the tumor, L and W are the lengths of the tumors, respectively. Also, the equation below was utilized to measure the inhibition rate (Abtahi et al., 2022):

$$Tumor\;inhibition\;rate\;\left(TIR,\;\%\right)=\frac{W_{c}-W_{t}}{W_{c}}\times 100$$

Where W_c_ and W_t_ represent the average weight of tumors in the control and treated groups, respectively.

### 2.14 Statistical analysis

Data were analyzed statistically using the GraphPad Prism6 software and reported as mean ± standard deviation. Student t-test was utilized to compare 2 independent groups, and multiple samples were compared by the ANOVA test. *p* values with less than 0.05, were considered as significant.

## 3 Results

Because of the widespread use of chemical pharmaceuticals and their negative side effects, interest in alternative therapies such as herbal medicines and gene therapy has grown. Herbal anticancer therapies have been pushed, including the isolation and identification of active plant components. New treatment tactics have recently switched to hybrid active agents, which are thought to be far more successful than separate forms. As a result of the reduced doses for each medicine, the treatment’s efficacy improves while adverse effects diminish. Curcumin, a yellow pigment extracted from the rhizome of the turmeric plant, has a wide range of medicinal benefits. Curcumin has also been demonstrated to have anticancer properties. It was recently incorporated as a chemical prophylactic factor in the first phase of clinical research. Curcumin may make cancer cells more responsive to several therapies, including chemotherapy and gene therapy, as a new anticancer agent. Curcumin’s therapeutic effectiveness, on the other hand, is hampered by its poor water solubility and, as a result, reduced therapeutic index. Nanotechnology is one of the powerful tools for increasing stability in aqueous solutions.

The therapeutic application of miRNAs has been documented in numerous research. However, their effectiveness is contingent on the carrier’s ability to transport them to cancer cells. MiRNA should be protected from nuclease degradation in an optimal transport carrier under systematic circulation. All requirements are endosomal escape, biocompatibility, renal and hepatic clearance resistance, and reversible physical binding to miRNAs. As previously said, niosomes are better than phospholipid vesicles because of their high drug loading efficiency, biocompatibility, biodegradability, low cost of manufacture, lack of organic solvents, easy storage, and superior stability. Herein, curcumin and miR-34a were separately loaded in DOTAP-containing cationic niosomal formulations. Tween-80, a safe and healthful surfactant, and Tween-60, a commonly used surfactant in niosome synthesis, were used to make the niosome vesicles. The curcumin and gene entrapment efficiency, particle charge, and size of each formulation were all evaluated. Release profiles were obtained by releasing the curcumin in different buffers. The optimal formulation was tested in cytotoxicity, cellular uptake, gene expression, apoptosis, and *in vivo* tests to assess their significance in cancer therapy.

### 3.1 Characterization of nanocarriers

#### 3.1.1 Curcumin entrapped niosomes: The effects of cholesterol on Curcumin’s EE%

Curcumin delivery systems are optimized based on several parameters, including the nanocarrier size, zeta potential, and entrapment efficiency (EE%, Table 1). Cholesterol is a key element of niosomes that impact their physicochemical properties and stability. The EE of nanocarriers increased from 35.78 to 48.45% when cholesterol content was increased from 10 to 20% (F1 and F2), the zeta potential was modified from - 24.88 to -22.43 mv, and the size of nanocarriers dropped from 134.12 to 129.34 nm. The type of nonionic surfactant appears to be a critical factor in the influence of cholesterol percentage on niosome size. It has been reported that the rate of average size reduction by adding cholesterol depends on the surfactant type (Alemi et al., 2018).

**TABLE 1:** The effect(s) of T-80:T-60:cholesterol ratio on the EE%, PDI, zeta potential (mV), and particle size (nm) in Cur-niosome particles.

Code	Cur (µg)	T-80 (%)	T-60 (%)	Cholesterol (%)	DOTAP (%)	EE (%)	PDI	Zeta potential (mv)	Particle size (nm)
F1	500	90	—	10	—	35.87	0.20 ± 0.45	−24 ± 0.88	134 ± 0.12
F2	500	80	—	20	—	48.45	0.19 ± 0.56	−22 ± 0.43	129 ± 0.34
F3	500	60	30	10	—	79.44	0.18 ± 0.23	−19.6 ± 0.67	114.1 ± 0.023
F4	500	60	25	10	5	93.45	0.16 ± 0.76	+4.44 ± 0.49	98.23 ± 0.03
F5	500	63	20	10	7	99.91	0.15 ± 017	+11.23 ± 0.02	87.23 ± 0.03

#### 3.1.2 The effects of T-60 and dioleoyl-3-trimethylammonium propane on Curcumin’s EE%

For investigating the effect of T-60 on the EE%, zeta potential, and size of nanocarrier, the cholesterol concentration was kept constant, and T-60 was (30%) added to the formulation (F3). The entrapment of curcumin within the niosomes raised to 79% and reduced the nanocarrier average diameter. Interestingly, EE% of curcumin exceeded 90% when DOTAP was introduced into the formulation (F4 and 5). DOTAP also enhanced the zeta potential, positively charged, and reduced the size of the particles. Therefore, it was concluded that the Cur-niosome preparations comprising Tween-80:Tween-60:cholesterol:DOTAP with the molar ratio of 63:20:10:7 (F5) owned the desirable properties based on the particle size and EE%. The DLS assay indicated that the average diameter of F5 was 87.23 ± 234 nm at a homogenized pressure of 800 bar. Moreover, including DOTAP in the formulation made the size reduction and helped the vesicles to maintain the drugs and displayed no remarkable alterations in 2 months of storing compared to other formulations with respect to vesicle size zeta potential and EE%.

#### 3.1.3 miR-34a entrapped niosomes

For preparing the miR-34a-niosome, almost the same material ratio used in F4, and F5 formulations of Cur-niosome, were employed. Because of their positive surface charge, positively charged lipids are frequently used to carry nucleic acids since these materials allow the spontaneous interconnection of negatively charged nucleic acids. The result is known as lipoplex. It has been shown that cationic lipids, such as 1,2-dioleoyl-3-trimethylammonium-propane (DOTAP), enhance positive loading on vesicles, making vesicles easier to connect with negative cell membranes *in vitro*. Due to their versatility, they are the most suitable gene carriers. In general, DOTAP-containing lipid vesicles have lower toxicities compared to those containing other kinds of lipids due to the inclusion of two ester linkages within the structure, says Balazs *et al.* (Balazs and Godbey, 2011). Likewise, with the addition of 10 and 15% DOTAP, particle size, charge, and zeta potential have changed. Therefore, it was concluded that the miRNA-niosome preparations comprising Tween-80:Tween-60:cholesterol:DOTAP with the molar ratio of 58:17:10:15 (F5) had the desirable property based on the zeta potential and particle size (Table 2).

**TABLE 2:** The effect(s) of T-80:T-60:cholesterol ratio on the PDI, zeta potential (mV), and particle size (nm) in miRNA-niosome particles.

Code	T-80 (%)	T-60 (%)	Cholesterol (%)	DOTAP (%)	PDI	Zeta potential (mv)	Particle size (nm)
F1	90	—	10	—	0.20 ± 0.45	−24 ± 0.88	134 ± 0.12
F2	80	—	20	—	0.19 ± 0.56	−22 ± 0.43	129 ± 0.34
F3	60	30	10	—	0.18 ± 0.23	−19.6 ± 0.67	114.1 ± 0.023
F4	60	20	10	10	0.168 ± 0.44	+13.04 ± 0.76	88.55 ± 0.44
F5	58	17	10	15	0.156 ± 0.23	+22.56 ± 0.11	81.45 ± 0.14
F6	F5 + miR-34a				0.173 ± 0.12	+8.23 ± 0.12	93.21 ± 0.34

### 3.2 Morphology of nanoparticles

**FIGURE 1:**
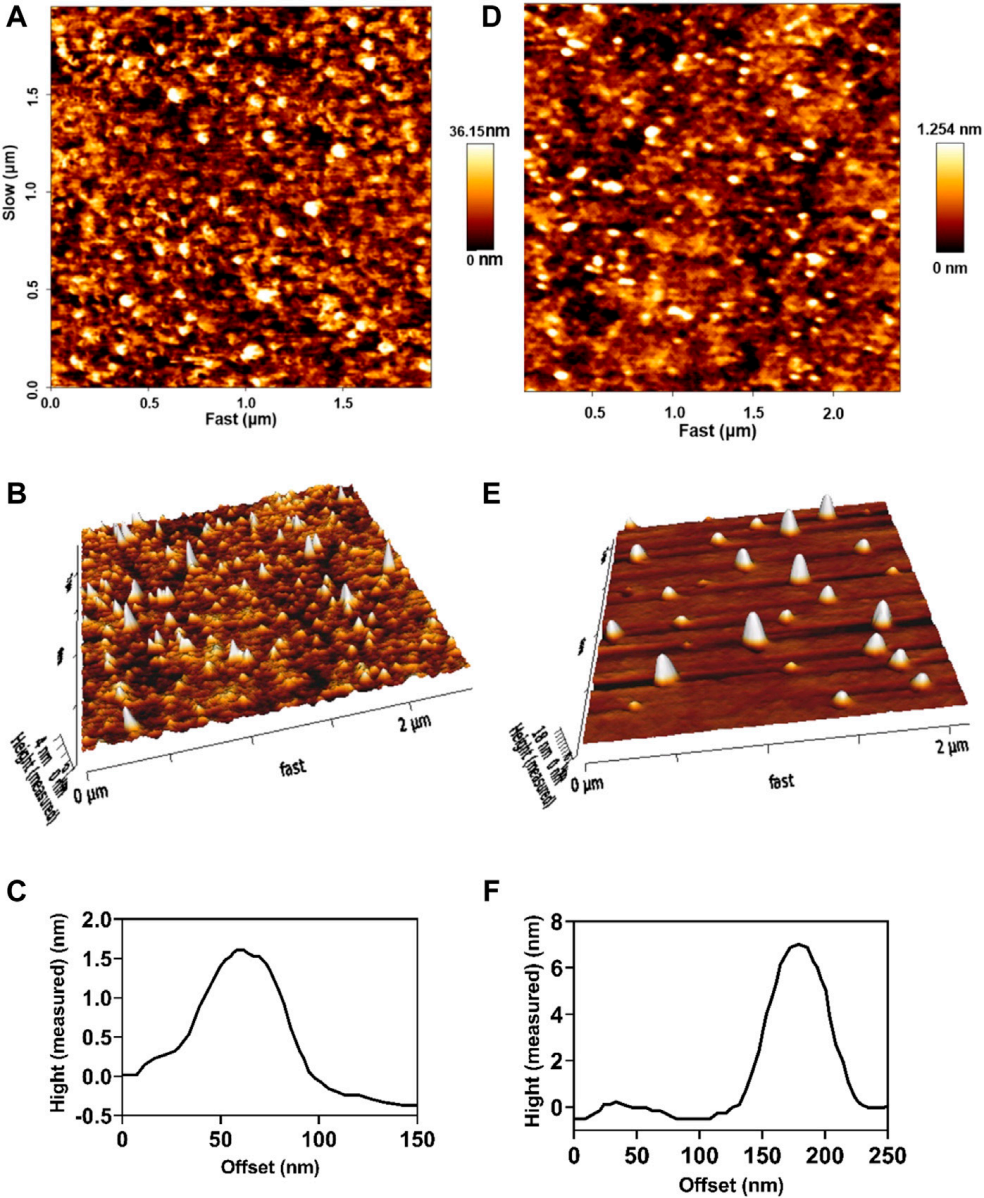
2D, 3D, and line analysis of Cur-niosomes as **(A–C)**, respectively. 2D, 3D, and line analysis of miRNA-niosomes as **(D–F)**, respectively.

**FIGURE 2:**
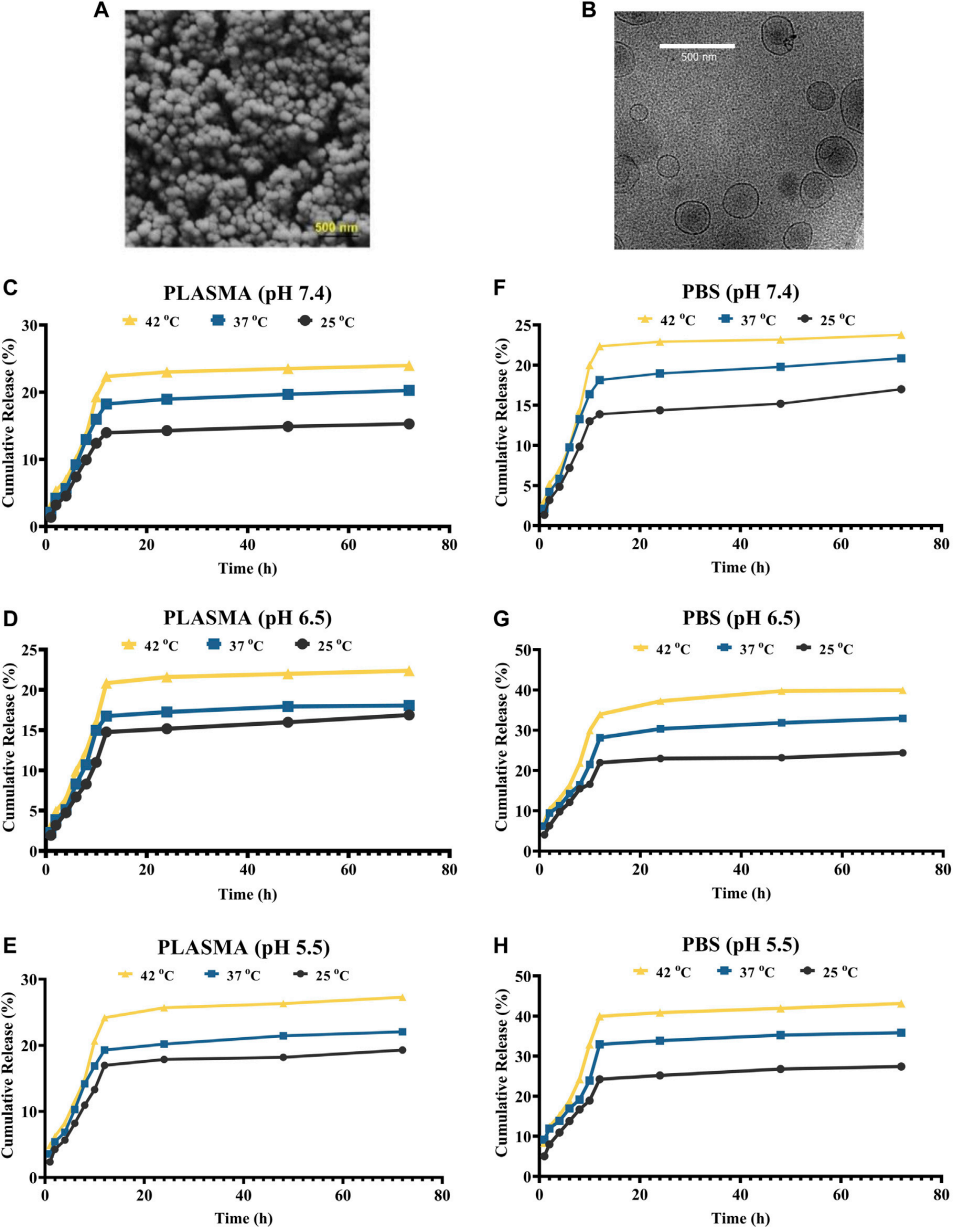
**(A,B)** SEM and cryo-TEM of the loaded niosomes, respectively. **(C–E)** Release of Cur in plasma solution at the pH of 7.4, 6.5, and 5.5, respectively. **(F–H)** Release of Cur in PBS solution at the pH of 7.4, 6.5, and 5.5, respectively.

The inner structure of drug-loaded niosomes was assessed by the AFM and shown in Figures 1A–F. The images were obtained from the optimal formula of niosomes in a sphere shape. SEM and TEM images were obtained in a similar way and shown in Figures 2A,B. In the images, niosome vesicles seemed to be spherical with smooth surfaces. The smooth surface provides the desired property for interaction with cells.

### 3.3 The *in-vitro* releasing pattern of cur-nanoparticles

The niosomes’ *in vitro* drug release patterns in plasma and PBS were studied at 25°C, 37°C, and 42°C at pH values of 5.5, 7.4, and 9 (Figures 2C–H). Plasma was utilized to explore the release experiment in a nearly human-like environment. By modifying the solubility of the delivery system or cleaving pH-sensitive bonds upon a pH gradient, pH-sensitive delivery systems can lead to a site-specific release of medicinal payloads. This pH gradient, which may be found in a variety of bodily locations (including the tumor environment, the gastrointestinal tract, the vaginal area, and blood arteries), can be exploited as an endogenous stimulus for developing smart delivery systems and regulating medication release. The extracellular pH of cancer cells is lower than that of normal cells, whereas the intracellular pH of cancer cells is greater. As a result, the drug’s release profile was studied under neutral, acidic, and alkaline pH environments. Every release profile is biphasic, with varying gradients. Cancer cells/tissues have a greater temperature than normal cells, which might be exploited to administer stimuli-responsive tumor-targeted drugs. At all of the pHs studied, the temperature influenced the release of curcumin from F5. For example, at pH 7.4, the release was 15.87, 19.84, and 23.76% at 25, 37, and 42°C in plasma, respectively. Furthermore, the data demonstrate that medication release is greater at acidic pH levels, which are typical of the tumor microenvironment than in neutral settings. Curcumin release from niosomal nanoformulation follows a two-phase pattern, according to *in vitro* release experiments. In the first phase, the medication is released quickly, followed by a slower release in the second phase. The dialysis bag or the medication size has no effect on the release. The initial rapid release is likely controlled by a diffusion mechanism caused by a curcumin gradient of concentration between the niosome and the buffer surrounding the dialysis bag, whereas the slow release in the second phase is caused by controlled release from the bilayer membrane of the niosomal formulation.

### 3.4 Physical stability assay

**FIGURE 3:**
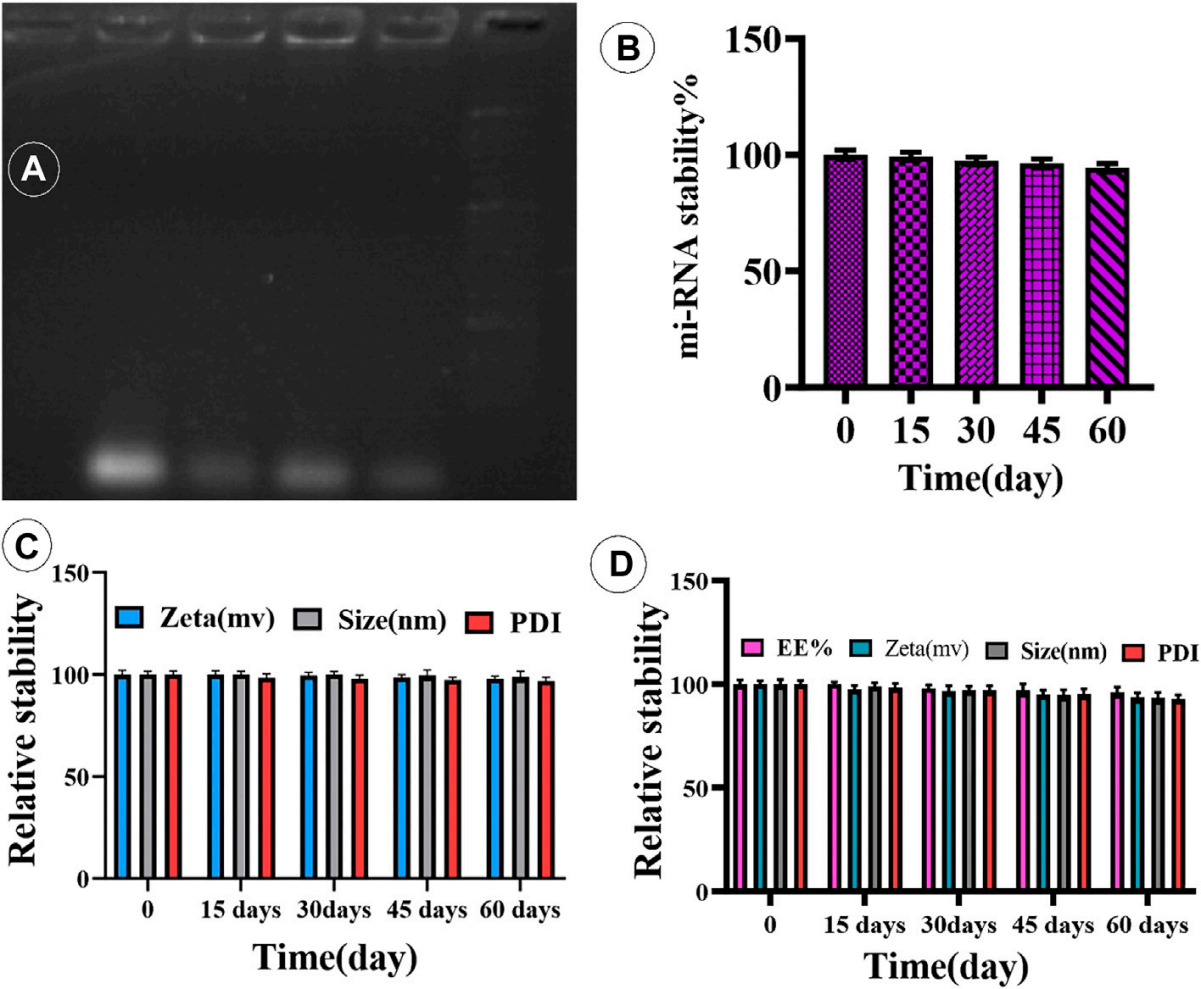
**(A)** Complex of miR-34a-loaded niosome. **(B)** Time-dependent stability of miR-34a-niosome. **(C,D)** Physical stability of Cur- and miRNA-niosome after 15, 30, 45, and 60 days, respectively.

Curcumin is a flammable, water-insoluble chemical. Curcumin was integrated into cationic niosomes to improve the drug’s stability and absorption. The drug-loaded nanocarrier’s great stability after protracted storage is critical for the drug’s effectiveness. The influence of particle size and zeta potential on vesicular system stability is well understood. As a result, barriers between vesicular particles are built to avoid accumulation. A charged molecule, for example, can be put on the system’s surface. The zeta potential is a charge indicator, and if the particles have the right zeta potential, they repel each other and do not combine. NCur (F5) was therefore kept at 4°C for 15, 30, 45, and 60 days. The zeta potential, vesicle size, PDI, and EE% were measured after each time interval. From the results in Figure 3, the size/PDI of the vesicles and the EE% of the optimal niosomal formulation (F5) showed no significant changes after 60 days of storage when compared to pristinely synthesized samples (*p*-value 0.05), indicating that NCur is physically stable for at least 60 days. Therefore, the inclusion of DOTAP in the niosomal formulations only affected the zeta potential and either vesicle with a positively or negatively charged surface (with or without DOTAP), did not show alternation in their stability after 15, 30, 45 and 60 days in storage.

### 3.5 Complex formation of niosome with miRNA

To confirm that cationic niosomes and miR-34a formed a compound, a gel retardation experiment was employed. Cationic niosomes bind miR-34a in a dosage-dependent manner, as seen in Figure 3B. As can be observed, the best condition was a ratio of 20:0.15 (cationic niosomes:mi-RNA34a). The loading of mi-RNAs onto cationic niosomes was ensured by zeta potential examination of the cationic niosomes before and after loading with miR-34a, as shown in Table 2. Furthermore, the size of the two parties grows due to electrostatic bonding and complex development. By forming a spatial barrier, the long chains of miR-34a that connect to the surface of niosomes directly enhance the hydrodynamic diameter. The final formulation (F5) has a size of 100 nm, which is satisfactory. The niosomes were stored at 4°C for 4 months to investigate the effect of long-term storage on miR-34a leaking from the surface. The complexes were stable with the lowest leakage after 4 months, according to the data (Figures 3C,D). The formulation is stable, according to the results of the stability test (electrophoresis), and the mi-RNA loaded in the niosome with a volume ratio of 20:0.15 (Niosome:mi-RNA) forms a complex.

### 3.6 Cytotoxicity assays

**FIGURE 4:**
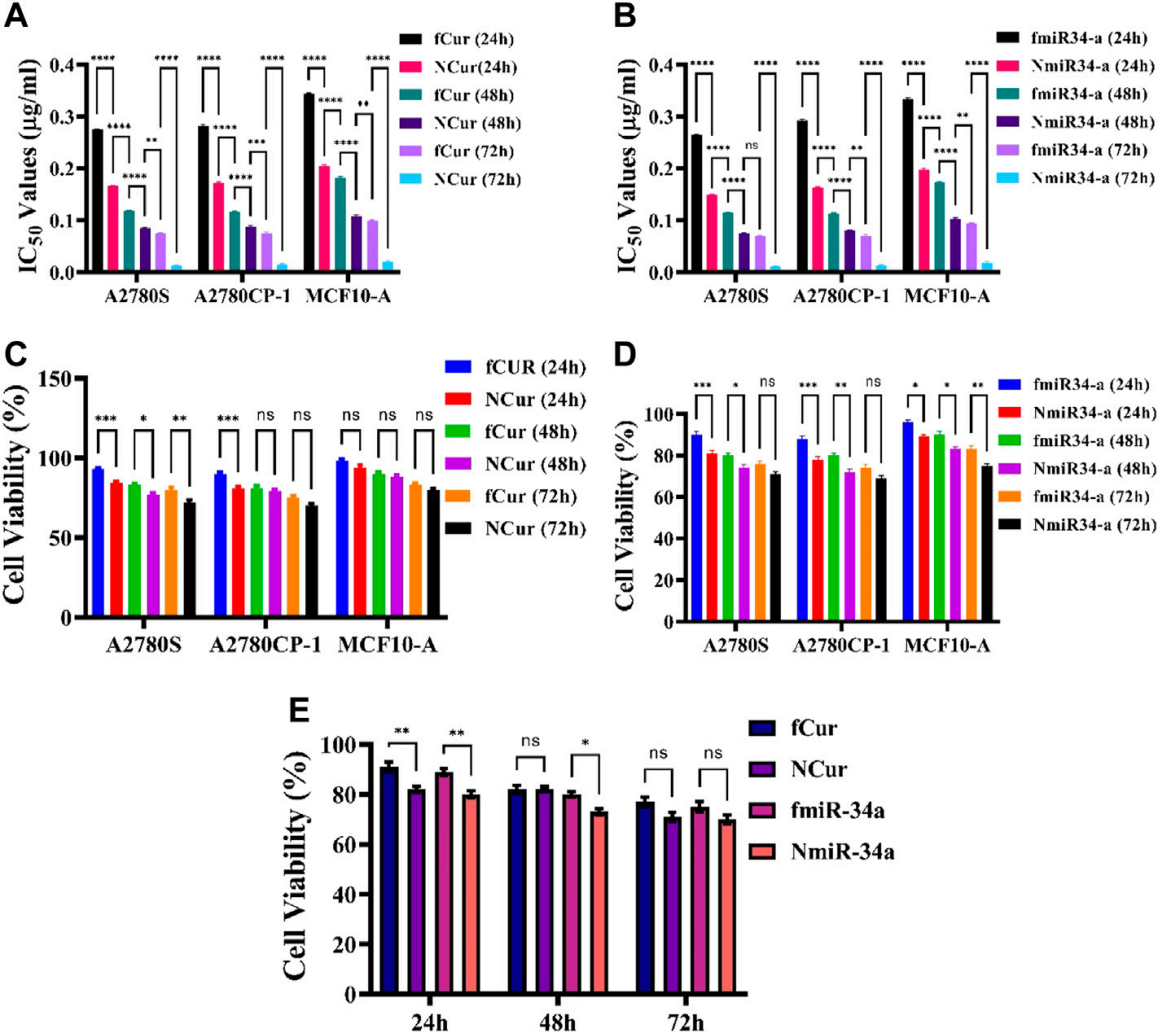
**(A,B)** IC_50_ values of free and niosomal Cur and miRNA on healthy and cancerous cells in 24, 48, and 72 h, respectively. **(C,D)** Cytotoxicity of free and niosomal Cur and miRNA on c healthy and cancerous cells in 24, 48, and 72 h, respectively. **(E)** A comparison between free and niosomal Cur and miRNA in 24, 48, and 72 h.

Initially, we looked at the effects of free and niosomal curcumin on the viability of normal (MCF10-a) and cancer cell lines (A2780S, A2780CP1). We did this by calculating the IC_50_ values after 24, 48, and 72 h. According to the findings, normal cells required higher concentrations of either fCur or NCur to achieve IC_50_ than malignant cells. Furthermore, the drug encapsulated in niosomes was observed to improve cytotoxicity as compared to fCur. In comparison with free Cur, the IC_50_ values for A2780CP-1 and A2780S cells treated with NCur decreased by 45.6, 34.8, and 34.1%, respectively when incubated for 24 h. After 72 h, the disparity in the IC_50_ values of cancer cells cultured with free and encapsulated medications grew significantly. To evaluate the cytotoxic activities of free and niosomal Cur and miRNA on the human ovarian cancer A2780s, A2780cp, and MCF10-A cell lines were measured using the MTT test after incubation of cells for 24, 48, and 72 h. First, the inhibitory effects of free and niosomal Cur on A2780s, A2780cp, and MCF10-A cells were determined by dose-response tests. Next, the IC_50_ levels of these substances were evaluated (Figures 4A,B). Results displayed higher concentrations of drugs for MCF-10A than A2780s and A2780cp cells to achieve IC_50_ value. In comparison with the free state, the utilization of niosomes demonstrated remarkably lower IC_50_ values for all the cell lines.

The observations showed that free Cur and Cur-niosome had less effect on the MCF10-A cell line. At an equal dosage, drugs with niosomal carriers showed higher cytotoxicity than free drugs (Figures 4C,D). A total comparison in the average of the mean values of all cell lines after 24, 48, and 72 h showed that there are significant differences in the first day between the free and niosomal forms. Although the differences reduce after 72 h, still the niosomal forms demonstrated higher toxicity than their free forms (Figure 4E).

### 3.7 Cellular uptake of niosomal systems

**FIGURE 5:**
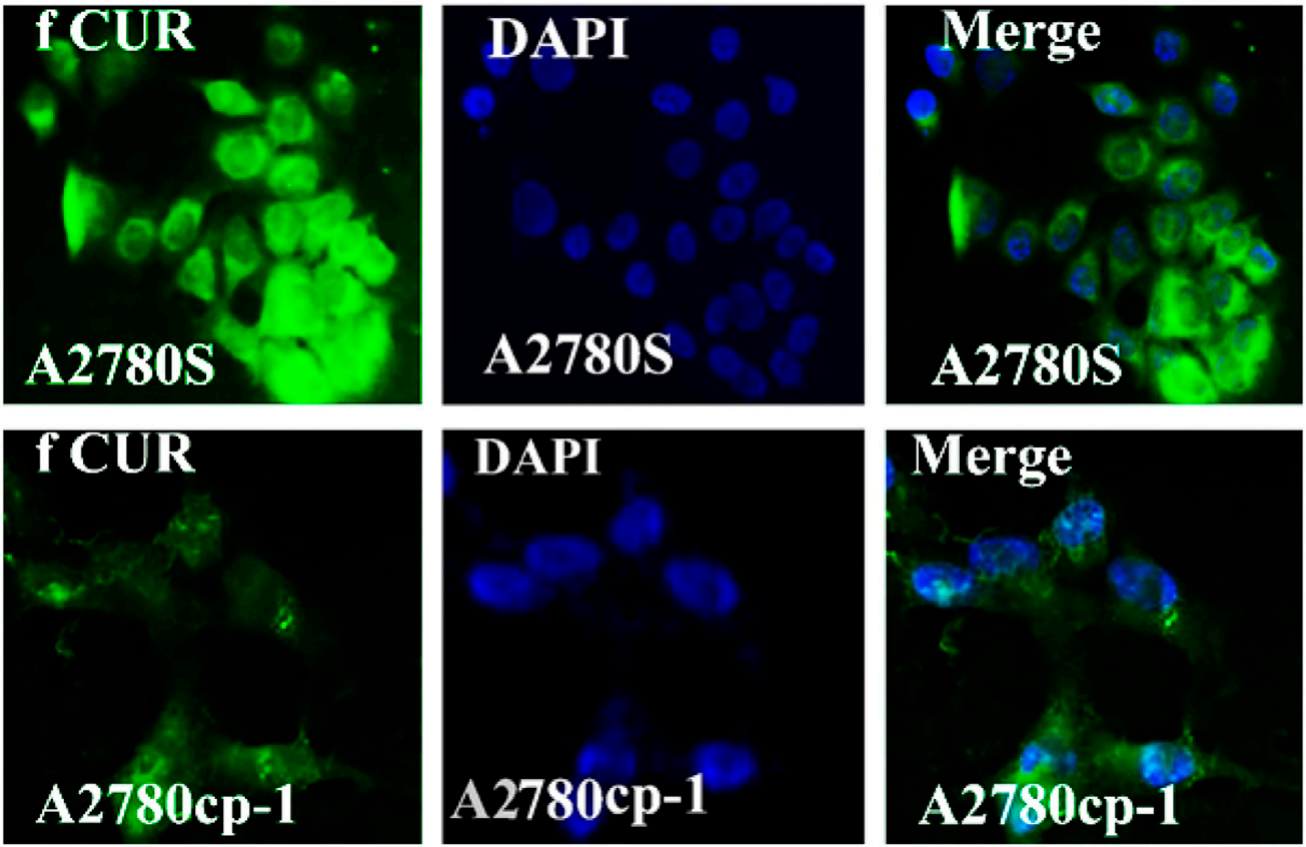
The uptake images of free Cur by A2780s and A270cp-1 cells after incubating for 4 h.

**FIGURE 6:**
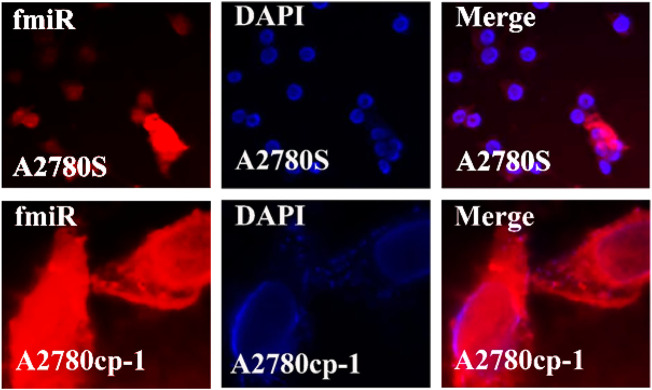
The uptake images of free miRNA by A2780s and A270cp-1 cells after incubating for 4 h.

**FIGURE 7:**
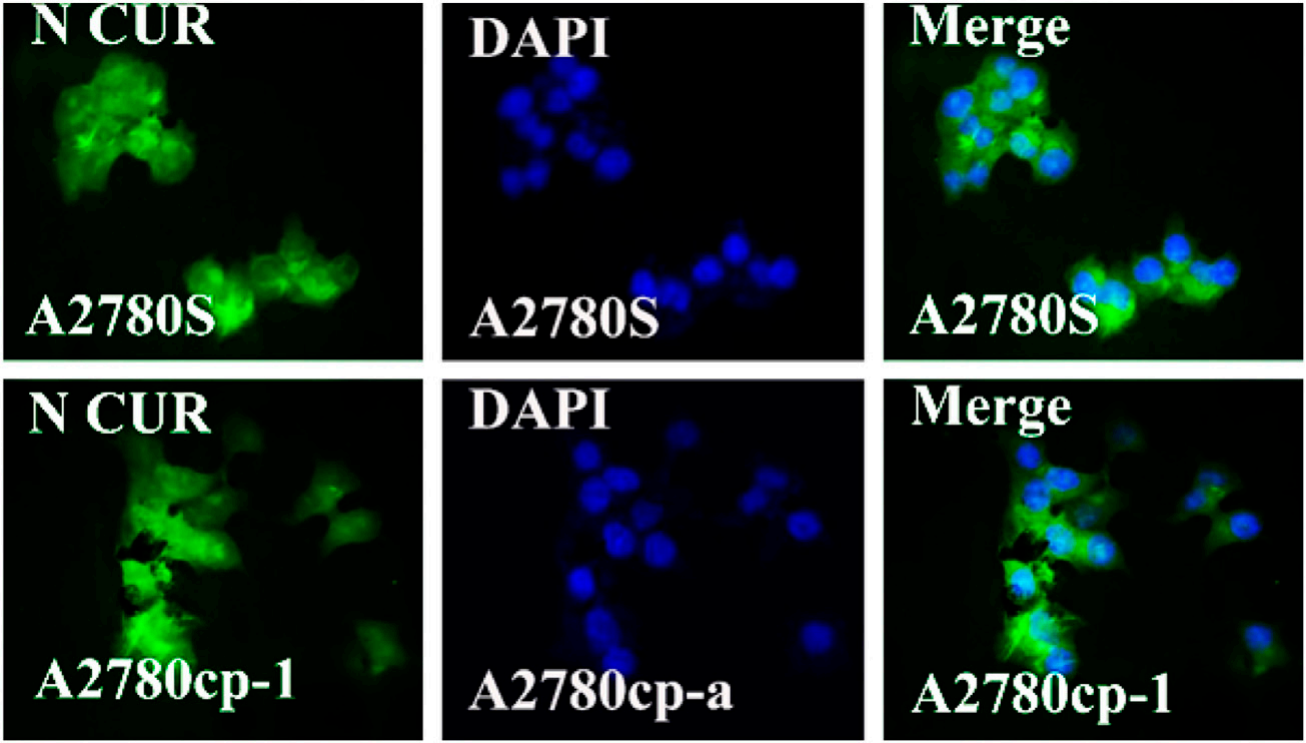
The uptake images of niosomal Cur by A2780s and A270cp-1 cells after incubating for 4 h.

**FIGURE 8:**
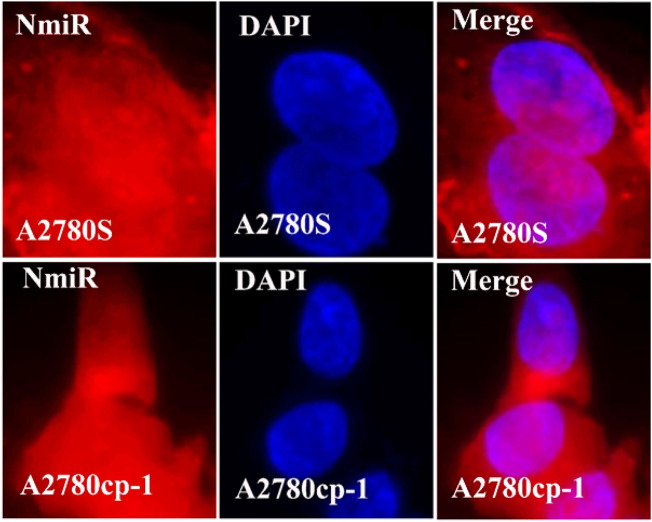
The uptake images of niosomal miRNA by A2780s and A270cp-1 cells after incubating for 4 h.

In the cellular uptake tests, cancer cell models A2780S and A270cp-1 were utilized (Figures 5, 6, 7, 8). The cellular uptake behavior of two niosomal formulations was examined. Fluorescence microscopy was used to capture pictures of cancer cell lines treated with unloaded niosome, fCur, fmiR, NCur, and NmiR (Figures 5, 6, 7, 8). Cells treated with fCur or NCur glow red, whereas cells treated with fmiR or NmiR fluoresce green. The fluorescence intensity of cells incubated with niosomal nanoformulations was higher than that of cells treated with free versions of the medication or gene. Furthermore, although miR-34a accumulated in the cytoplasm, curcumin accumulated in both the nucleus area and the cytoplasm. Cancer cells are found to effectively absorb cationic niosomes. Endocytosis plays a significant role in the penetration of drug-loaded niosomes into cells as well as the transport of curcumin and miRNA into them as compared to free medicines transferred through the cell membranes *via* a diffusion process. Niosomes enhanced the solubility of curcumin, which has been considered as a hydrophobic medicine.

### 3.8 Gene expression

To measure the gene silencing efficiency of Cur and miR-34a, the expression of Nf-ĸB and P53 was evaluated to confirm their downstream impact. Generally, the anti-cancer mechanism of Nf-ĸB is based on the mitochondrial apoptosis pathways. Herein, qRT-PCR techniques were employed to detect the expression of Nf-ĸB and P53. For this purpose, first, cells were subjected to free Cur and miRNA, as well as niosomal Cur and miRNA, then, the expression of the genes was measured. The results showed a minor reduction in NF-κB and major improvement in P53 gene expression compared to the control group. Moreover, using the niosomal carrier remarkably enhanced the expression compared to their free forms. Specifically, it was observed that the gene expression using miRNA was higher than Cur. Ultimately, the gene expression of P53 and Nf-ĸB can be effectively improved by the utilization of nanocarriers by facilitating the intracellular delivery of medicines. The same results were achieved by both cytotoxicity and apoptosis assays.

### 3.9 Apoptosis analysis

**FIGURE 9:**
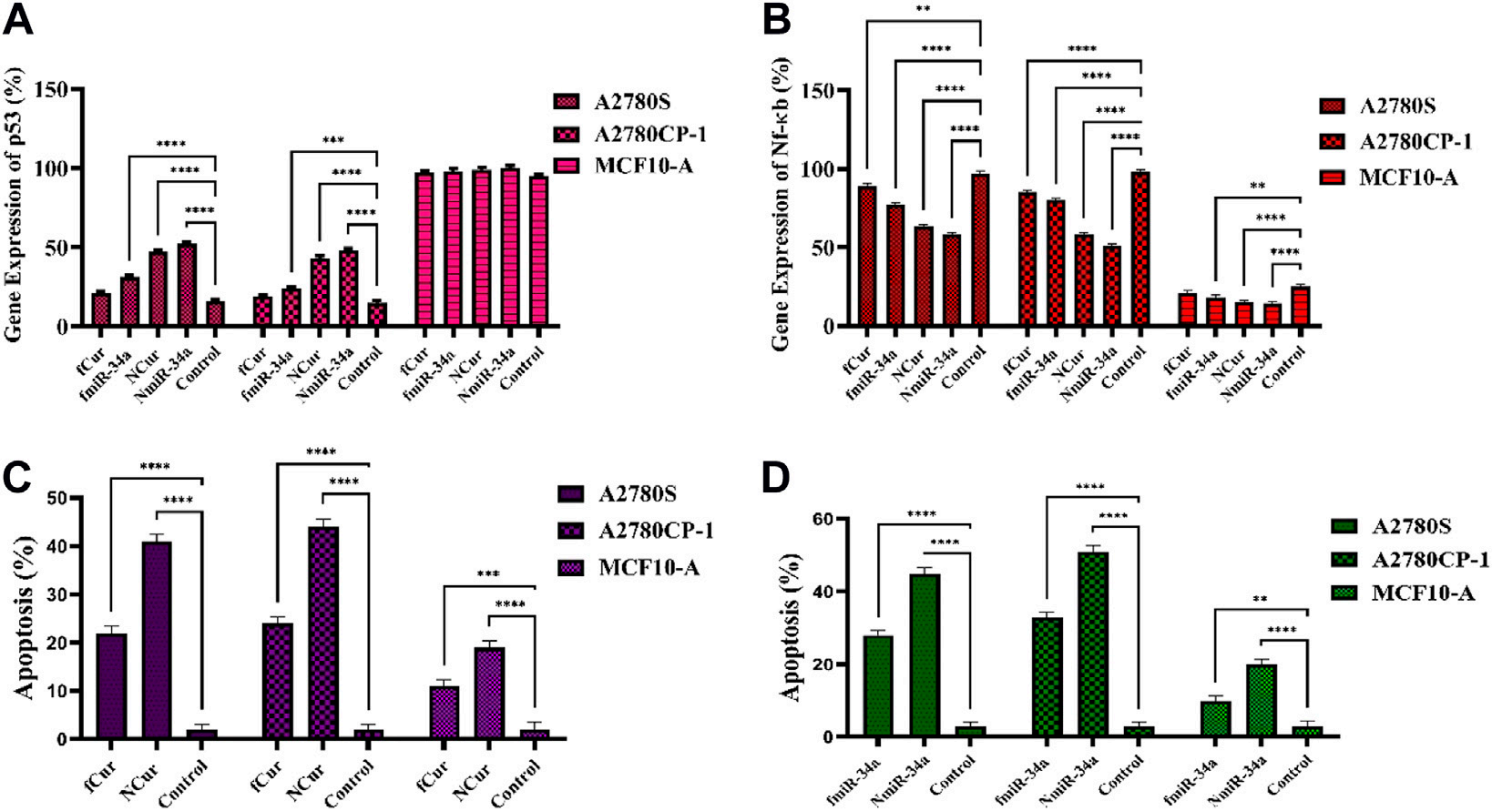
**(A,B)** The real-time PCR analysis of the expression of p53 and Nf-ĸB genes subjected to the samples for 48 h **(C,D)** Apoptosis assay of cells subjected to the samples using flow cytometry after 24 h.

To perform the apoptosis test on the cells, Annexin V-FITC/PI double staining assay was utilized on free Cur, free miRNA, niosomal Cur, and niosomal miRNA for 24 h and the result was obtained using a flow cytometer. Generally, in this assay, the phospholipid phosphatidylserine (PS) of the cell membrane of apoptotic cells translocates from the inward to the outward surface of the plasma membrane and subjects the PS to the exterior cellular environment. Annexin V is a Ca^2+^-dependent phospholipid-binding protein with 35–36 kDa which has a high affinity to bind with PS of apoptotic cells. To improve the detection while maintaining the high affinity to PS, it is possible to conjugate annexin V to the fluorescent molecule like fluorescein isothiocyanate (FITC) and synthesis of Annexin V-FITC. Thus, to conduct the flow cytometric analyses, Annexin V-FITC was utilized which is a highly reactive probe for measuring the cells apoptosis. Moreover, to stain DNA, propidium iodide can be utilized not only is it a fluorescent DNA intercalating agent, but it also cannot penetrate inside the live or early apoptotic cells. Ultimately, the results were recorded after treatment for 48 h and displayed in Figure 9. It was observed that the utilization of niosomal carriers could effectively increase the apoptosis rate compared to their free forms. Moreover, the impact of miR-34a on cells in both forms was relatively higher than Cur.

### 3.10 *In-vivo* tumor prohibition

**FIGURE 10:**
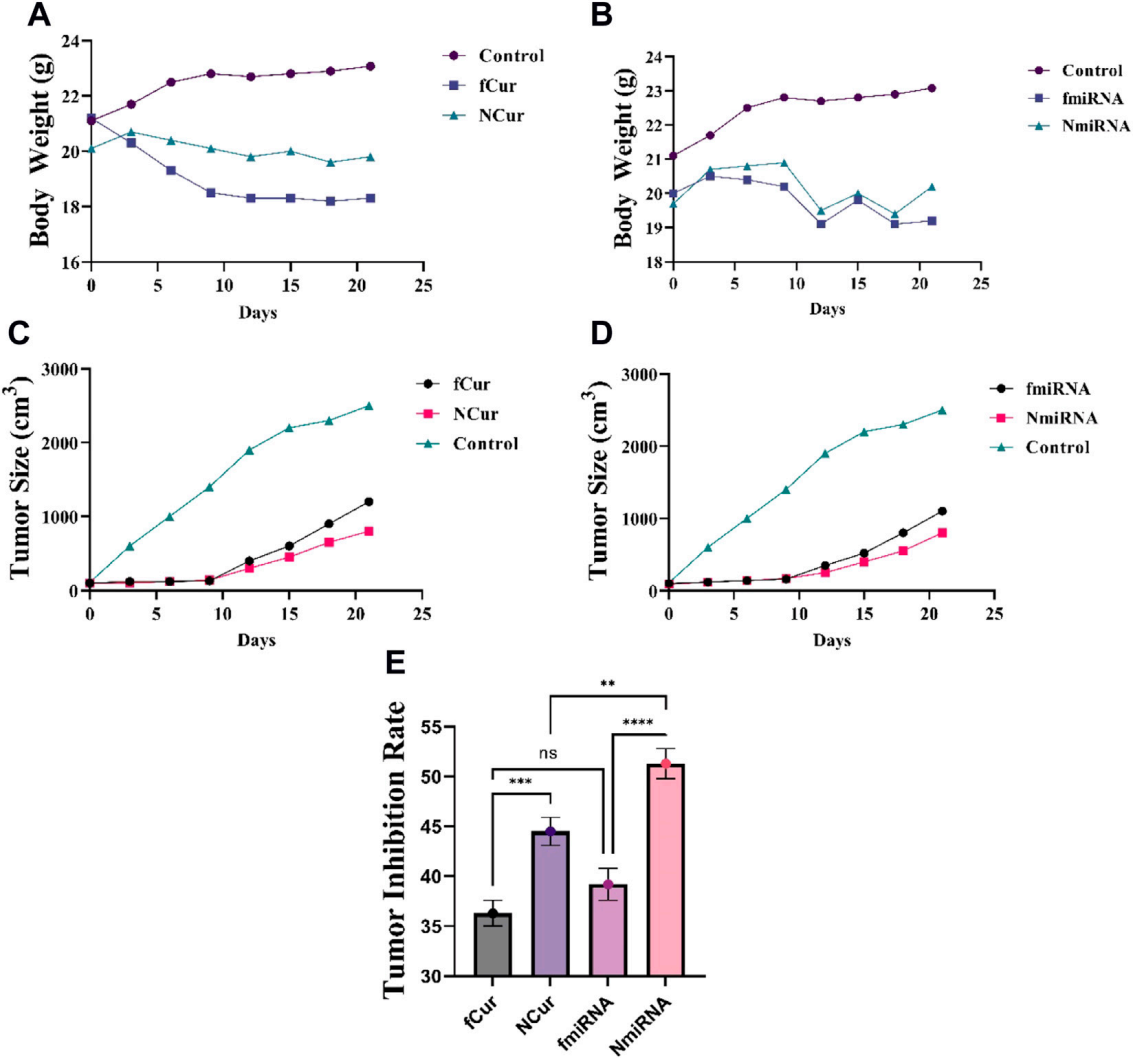
**(A,B)** Effect of Cur and miRNA formulations on the bodyweight at every 3-day-intervals, respectively. **(C,D)** The tumor size (cm^3^) of different mice groups after 21 days of receiving the medicine. All groups displayed a major decrease (*p* < 0.05) in the tumor volumes than the control group after 14 and 21 days. **(E)** Tumor inhibition rate.

To assess the antitumor activity of the formulations, 4T1 xenografted Balb/C mouse tumor models were conducted. The mice from each group were subjected to one medicine including free Cur, free miRNA, niosomal Cur, and niosomal miRNA, as well as normal saline, every 3 days until 12 days. Medicins were injected into the tail vein of the mice. After 21 days, to evaluate the tumor volumes after the sacrifice and removal, a digital vernier caliper was employed. The results demonstrated that free Cur and miRNA groups displayed a reduction in the tumor size compared to the saline group. Likewise, niosomal Cur and miRNA groups had even smaller tumors than the previous groups (Figure 10E). It can be concluded that the decrease in cell growth was the result of the constant inhibition by the samples. The data concluded that niosomal miRNA could enhance the tumor prohibition activities and also, it presented an augmented therapeutic impact on cells more than that of free miRNA or free forms of Cur and miR-34a (Figures 10C,D). The cytotoxicity of all medicine was assessed by weighing the mice at each interval (Figures 10A,B; Table 3). The body weight of treated mice encountered a minor reduction after 21 days, whereas the saline group gained weight. Meanwhile, on the 6th day, Cur groups faced the highest and the niosomal miRNA the lowest reduction in the bodyweight compared to the other groups.

**TABLE 3:** The measured bodyweight (g) of the mice after receiving different medicine.

Duration (day)	Control group	Free Cur	Free miRNA	Niosomal Cur	Niosomal miRNA
0	22.1	22	22	22.1	22
3	22.7	21.1 ± 0.1	21.2	21.7	21.8
6	23.5 ± 0.1	20	20.7 ± 0.1	21.1	21.4 ± 0.01
9	23.8	19.3	20.1	20.6	21.2
12	23.7	19.1 ± 0.1	20 ± 0.1	20.1 ± 0.1	20.8
15	23.8 ± 0.1	19.2	20	20	20.8 ± 0.01
18	23.9	19.3 ± 0.1	19.8 ± 0.1	20.1 ± 0.1	20.6
21	24 ± 0.2	19.4	19.8	19.8 ± 0.1	20.2 ± 0.1

## 4 Discussion

Chemotherapy is rapidly becoming the primary mode of treatment for a wide range of malignancies. The key issues in this context are the level of safety and the clinical efficacy of drugs. However, the use of chemical compounds has side effects such as toxicity, weight loss, and limited therapeutic efficiency.

Further research on herbal medication, either alone or in combination with chemotherapies, has recently shown its promise for cancer therapy. Curcumin, a yellowish spice obtained naturally from the rhizome of Curcuma longa, has been utilized for millennia in Asian nations and has been shown to have a wide variety of pharmacologic effects. This powder has antitumoral properties by inhibiting cell cycle signaling pathways, resulting in cell death. Cur has been shown in several studies to be effective against various cancer cell lines, including cervical, oral epithelial, brain, breast, hepatic, leukemia, colon, ovarian, pancreatic, melanoma, gastric, and prostate (Syng-Ai et al., 2004; Yallapu et al., 2013; Ag Seleci et al., 2016). Despite its impressive footprints, Cur’s wide therapeutic benefits are restricted due to its weak water solubility and fast metabolism. To address this issue, nanotechnology developed nano DDSs including vesicular nanocarriers and niosomes. Surfactant and cholesterol are combined to generate unilamellar or multilamellar vesicles that contain both hydrophobic and hydrophilic drugs. As a result, the use of DDSs boosted medication efficacy, chemical stability, lowered economic preparation costs, and simplified long-term storage (Feng, 2004; Sezgin-Bayindir and Yuksel, 2012; Yallapu et al., 2012).

To encapsulate Cur, a new cationic niosomal preparation was designed in the present research. The vesicular system was made using T-80 and T-60 as the most frequently used surfactants in the new formulation of niosomes and industrial surfactants, respectively. The introduced formulations were analyzed by their entrapment effectiveness, zeta, drug release, and vesicle size. The size of nanoparticles has significant effects on the physical stability, cellular intake, and drug release from nanoparticles (Mukerjee and Vishwanatha, 2009). The sizes of nanoparticles in this study were between 87 and 135 nm compared to the other study which they were ranged from 50 to 500 nm, with different Cur formulations indeed (Ravindran et al., 2009). The zeta potential of the Cur preparation was +11.23 mV, which guaranteed physical stability and prevented aggregation during long-term storage (Garces et al., 2015). The investigation of the nano-vesicle morphology images (AFM and SEM) displayed spherical and circular particles with flat faces. After prolonged storage of 60 days, the result did not show any significant changes in the physiochemical features of the encapsulated drug inside the nanoparticles compared to the original samples. *In-vitro* treatment with Cur in both niosomal and free forms was exerted on A2780s and A2780cp cell lines, as the cancer cells and MCF10A cells, as control cells by a standard MTT assay. The results showed that Cur-contained nanoparticles had lower IC_50_ values on cancer cells than free Cur. Furthermore, cancer cells have reduced vitality than normal cells when treated with niosomal Cur. By activating growth inhibitory pathways in tumor cells, Cur-loaded nanoparticles might downregulate growth factors and their receptors, including NF-B activity, signaling channels for PI3K-AKT activity, and therefore block cancer cell development (Wang J et al., 2016). As a consequence of the gene expression data, free Cur had a similar impact on A2780s and A2780cp cells as cancer cells, but its adverse effects on normal cells were significantly lower. Furthermore, fluorescence microscopy on intracellular accumulation was explored, and it revealed that cancer cell absorption of Cur was simpler than free Cur. Finally, it was determined that the absorption of niosomal Cur by cancer cells *in vitro* may boost therapeutic optimism as a potential cancer therapy approach (Ravindran et al., 2009).

MicroRNA-34a (miR-34a) is a miRNA that is transcriptionally controlled by the p53 network and has been found to be significantly downregulated in a number of malignancies. In triple-negative and mesenchymal-type breast cancer cell lines, miR-34a expression has been found to be lower. Through targeting Bcl-2, CD44, and SIRT1 (silent information regulator 1), Rac1, Fra-1, Notch-1, and different cyclins, exogenous expression of miR-34a in breast cancer cells caused cell death and decreased cell proliferation and migration. For miRNA delivery, a number of viral carriers have been developed, with good transfection effectiveness across a wide range of cell types. However, safety problems are now seen as a barrier to viral vector-based therapy’s practical implementation. Many kinds of nanoparticles have been utilized in gene delivery and transfection because of their small size, surface charge, and high surface area. In this study, DOTAP prepared the positive surface charge to link the miR-34a gene electrostatically on the surface of nanoformulation. Utilizing electrostatic interactions for loading the genes are the facile and conventional methods in nanoformulation when the controlled release is matter. Deng *et al.* fabricated a miR-34a containing nanoformulation using chitosan, hyaluronic acid, and doxorubicin for codelivery of doxorubicin and miR-34a against triple-negative breast cancer (Deng et al., 2014). They found that molecular weight and electrostatic interaction density of miR-34a strongly influenced the release profile of genes and drugs. The employment of suitable materials such as DOTAP, Tween-60, Tween-80, and cholesterol a, which have already been used in drug delivery applications, is a point of strength in our inquiry regarding the production of nanomaterials for the effective distribution of miR-34a. These nanomaterials produced an efficient anticancer impact against ovarian carcinoma *in vitro* and 4T1 breast cancer model *in vivo* by way of intracellular localization of miR-34a, as established *in vitro*. The finding of the present study was in line with our previous reports and other research on niosome-based systemic drug delivery.

## 5 Conclusion

Ovary cancer is a primary cause of mortality in gynecological malignancies, which can be treated with surgery, chemotherapy, and radiation therapy. The majority of ovary cancers have acceptable initial responses to the existing therapeutics. One of the suitable approaches for the treatment of present ovary cancer is the use of chemo/radio-sensitizer accompanied by chemo/radiation methods. Hence, curcumin has earned the attention of researchers in the treatment of malignancies with unique properties such as anti-apoptosis, anti-inflammation, anti-angiogenesis, and chemo-sensitivity. Nevertheless, the weak aqueous solubility and fast metabolism of curcumin significantly restricted its clinical application. Using a vesicular nanocarrier, e.g., niosomes, which are a substitute for phospholipid vesicles for encapsulating hydrophobic drugs, helped to treat cancers due to the provision of high encapsulation efficiency, biocompatibility, biodegradability, low formulation costs, and adequate stability. Also, such nanocarriers are devoid of organic solvents and can be stored easily. Herein, we synthesized a curcumin-contained niosomal system, specifically designed for the effective delivery of curcumin to treat ovary cancer. According to the results, niosomal protection improved biochemical stability and elevated the entrapment efficiency of curcumin. Besides, it demonstrated improved cellular intake and cytotoxicity against the ovarian cancer cells, A280s, and A280cp-1. Altogether, curcumin-niosomes have the potential to be a suitable delivery system for curcumin to combat ovary cancer. The cellular toxicity of miR-34a-niosome was enhanced in cancer cells, compared to free miR-34a. Likewise, the antitumor influence of gene delivery was studied *in vivo*. According to the *in vitro* and *in vivo* results, gene delivery from the developed nanoscaled niosomes was more successful than curcumin delivery. Therefore, gene therapy using niosomal particles was a favorable strategy for advanced cancer therapy.

## Data Availability

The raw data supporting the conclusion of this article will be made available by the authors, without undue reservation.
